# Phylloseptin-PBa—A Novel Broad-Spectrum Antimicrobial Peptide from the Skin Secretion of the Peruvian Purple-Sided Leaf Frog (*Phyllomedusa Baltea*) Which Exhibits Cancer Cell Cytotoxicity

**DOI:** 10.3390/toxins7124878

**Published:** 2015-12-01

**Authors:** Yuantai Wan, Chengbang Ma, Mei Zhou, Xinping Xi, Lei Li, Di Wu, Lei Wang, Chen Lin, Juan Chavez Lopez, Tianbao Chen, Chris Shaw

**Affiliations:** 1 Natural Drug Discovery Group, School of Pharmacy, Queen’s University, Belfast BT9 7BL, Northern Ireland, UK; ywan01@qub.ac.uk (Y.W.); c.ma@qub.ac.uk (C.M.); m.zhou@qub.ac.uk (M.Z.); xxi01@qub.ac.uk (X.X.); lli12@qub.ac.uk (L.L.); dwu03@qub.ac.uk (D.W.); t.chen@qub.ac.uk (T.C.); chris.shaw@qub.ac.uk (C.S.); 2 College of Basic Medical Science, Zhejiang Chinese Medial University, Hangzhou 310053, China; 3 Perubiotech Eirl, Santiago de Surco, Lima 33, Peru; perubiotech@gmail.com

**Keywords:** amphibian, antimicrobial, anticancer, peptides, molecular cloning, mass spectrometry

## Abstract

Antimicrobial peptides from amphibian skin secretion display remarkable broad-spectrum antimicrobial activity and are thus promising for the discovery of new antibiotics. In this study, we report a novel peptide belonging to the phylloseptin family of antimicrobial peptides, from the skin secretion of the purple-sided leaf frog, *Phyllomedusa baltea*, which was named Phylloseptin-PBa. Degenerate primers complementary to putative signal peptide sites of frog skin peptide precursor-encoding cDNAs were designed to interrogate a skin secretion-derived cDNA library from this frog. Subsequently, the peptide was isolated and identified using reverse phase HPLC and MS/MS fragmentation. The synthetic replicate was demonstrated to have activity against *S. aureus*, *E. coli* and *C. albicans* at concentrations of 8, 128 and 8 mg/L, respectively. In addition, it exhibited anti-proliferative activity against the human cancer cell lines, H460, PC3 and U251MG, but was less active against a normal human cell line (HMEC). Furthermore, a haemolysis assay was performed to assess mammalian cell cytotoxicity of Phylloseptin-PBa. This peptide contained a large proportion of α-helical domain, which may explain its antimicrobial and anticancer activities.

## 1. Introduction

The skins of frogs secrete a profusion of bioactive peptides, especially antimicrobial peptides, which function in the killing of bacteria and their biofilms. In the 30 years since the discovery of the first antimicrobial peptide (AMP) from this source, hundreds of such peptides have been identified [1]. *Phyllomedusinae* is a sub-family of the *Hylidae*. It consists of 57 species in seven genera: *Agalychnis, Cruziohyla, Hylomantis, Pachymedusa, Phasmahyla, Phrynomedusa* and *Phyllomedusa* [2] which are widely distributed across Central and South America. The skin secretions of phyllomedusine frogs have been found to produce a large number of AMPs with various antimicrobial activities. As a consequence of their structural similarities and secondary structural characteristics, these AMPs have been classified into six families [3,4]. The peptides from different families have distinctive primary structures. However, their biosynthetic precursors display strong evolutionary homologies, particularly in their highly conserved signal peptide and *N*-terminal pro-regions [5].

Phylloseptins are a family of AMPs recently discovered from the skin secretion of *phyllomedusine* frogs. They were first isolated from the skin secretion of *Phyllomedusa* *hypochondrialis* and *Phyllomedusa oreades* by Leite in 2005 [6]. As time passed, more phylloseptins were isolated and identified from other species. The biosynthetic precursors of these phyllposeptins exhibited common characteristic structures and shared highly-conversed domains within their precursor-encoding cDNAs [7]. The primary structure of phylloseptins usually comprises 19–21 amino acid residues and they are positively charged, *C*-terminally amidated, and additionally contain a α-helical domain. The natural characteristic of amphiphilicity of these peptides was proposed to account for their bioactivity via combination with cytoplasmic membranes, prior to their disruption. Since they display little haemolytic activity, phylloseptins are regarded as preferentially targeting prokaryotic rather than eukaryotic membranes [6,7].

In this study, a novel phylloseptin, named Phylloseptin-PBa, was isolated from the skin secretion of *Phyllomedusa baltea*—the first report of phylloseptin peptides from this frog. The structure of the peptide was obtained via “shotgun” cloning using 3′RACE and 5′RACE. The predicted primary structure was confirmed by MS/MS fragmentation sequencing. The synthetic replicate of the peptide was subjected to antimicrobial, anticancer and haemolysis assays in order to determinate its biological function. Phylloseptin-PBa displayed inhibitory activity against *E*. *coli*, *S. aureus* and *C. albicans*. Specifically, the growth of the standard Gram-positive bacterium, *S. aureus*, was significantly inhibited by Phylloseptin-PBa, but the peptide also inhibited the pathogenic yeast, *C*. *albicans*, at the same minimal inhibitory concentration (MIC). A further study was carried out to determine the concentration of Phylloseptin-PBa at which no growth of *S. aureus* and *C. albicans* occurred and this was found to be 8 mg/L in both cases. However, Phylloseptin-PBa was not active against *E*. *coli* at concentrations up to 128 mg/L. It also possessed a relatively low potency of haemolysis at concentrations that were effective against *S. aureus* and *C. albicans*. Meanwhile, the anticancer activity assay of this peptide indicated that it was active on all three human cancer cell lines tested (H460, PC3 and U251MG) but was less active on a normal human microvessel endothelial cell line (HMEC-1).

## 2. Results

### 2.1. Shotgun Cloning of Novel Peptide Precursor-Encoding cDNA

**Figure 1:**
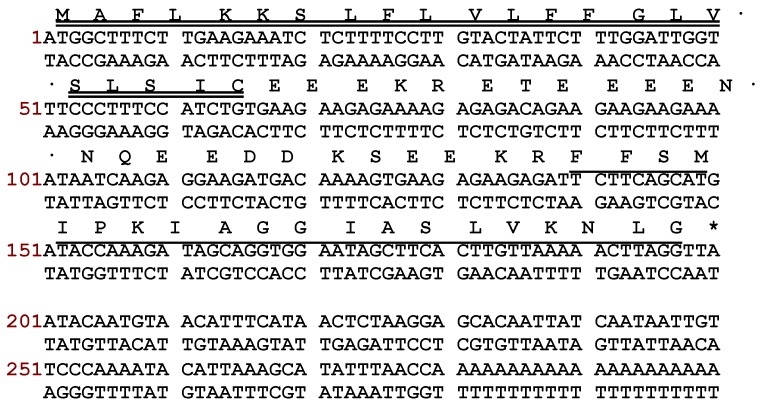
Nucleotide and translated open-reading frame amino acid sequences of cloned cDNA encoding the biosynthetic precursor of Phylloseptin-PBa. Double underlining indicates the putative signal peptide sequence, single underlining indicates the mature peptide sequence and an asterisk indicates the stop codon.

**Figure 2:**
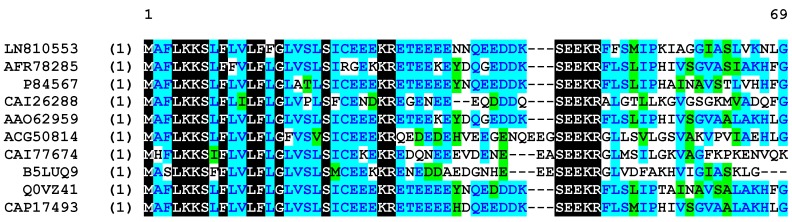
Multiple alignment of the cloned cDNA-deduced amino acid sequence of Phylloseptin-PBa obtained in this study, with those of the top hits following NCBI-BLASTp analysis. Gaps have been included to maximize alignments. Black shading indicates identical amino acid residues among all the sequences, blue shading indicates consensus amino acid residues, and green shading indicates similar amino acid residues.

Using a *Phyllomedusinae*-specific degenerate primer, designed to the highly conserved signal region from previously sequenced peptides from *phyllomedusine* frogs, a full-length cDNA encoding Phylloseptin-PBa was successfully and repeatedly cloned (at least 25 clones were represented) from the skin secretion-derived cDNA library of *Phyllomedusa baltea*. Through bioinformatic searches, the putative mature peptide was analysed using the NCBI BLASTp program, which demonstrated that Phylloseptin-PBa was a new member of the phylloseptin family. The nucleotide and translated open reading frame amino acid sequences of precursor-encoding cDNA are shown in Figure 1. Essentially, there were several defining characteristics of note: (1) the open reading frame of the precursor consisted of 66 amino acid residues, which containing the mature peptide of 19 amino acids; (2) A highly- conserved putative signal peptide region of 21 amino acid residues; (3) An acidic amino acid residue-rich “spacer” peptide containing 22 residues; (4) A classical propeptide convertase processing site (-KR-); (5) A mature active peptide encoding domain that contained 19 amino acid residues with a typical phylloseptin *N*-terminal; (6) A *C*-terminal G residue that acts as an amide donor for the L residue that terminates the mature peptide. According to the results of BLAST analyses, Phylloseptin-PBa showed a high level of structural homology to phylloseptins from other frogs of the sub-family, *Phyllomedusinae*, including PS-7 to PS-11 from *Phyllomedusa hypochondrialis*, PS-7, 8, 12 and 15 from *Phyllomedusa azurea*, PS-1 from *Phyllomedusa sauvagei* and PS-B from *Phyllomedusa bicolor* [3]. The alignment of amino acid sequences of the precursor isolated from *Phyllomedusa baltea* with the top hits found in the database, are shown in Figure 2. The nucleotide sequence of the Phylloseptin-PBa precursor has been deposited in the European Molecular Biology Laboratory (EMBL) Nucleotide Sequence Database under the accession code LN810553.

### 2.2. Isolation and Structural Characterization of Phylloseptin-PBa from Reverse Phase HPLC Fractions of Skin Secretion

**Figure 3:**
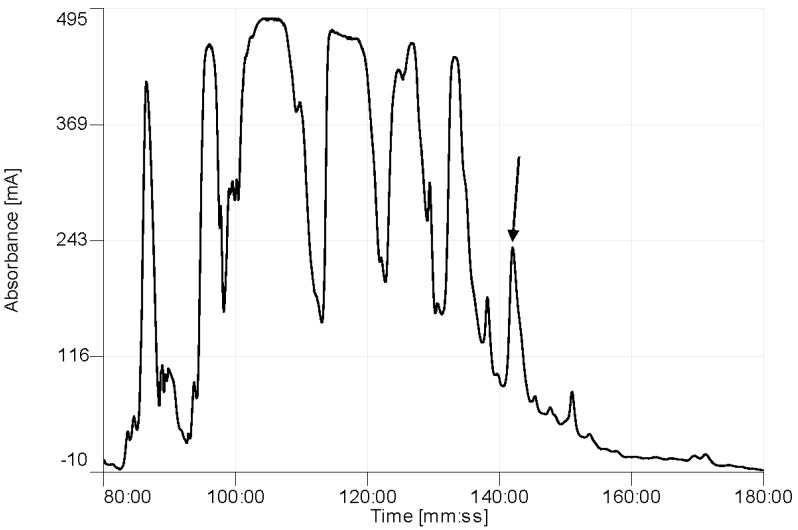
Region of reverse phase HPLC chromatogram of *Phyllomedusa baltea* skin secretion showing the absorbance peak (arrow) corresponding to Phylloseptin-PBa. The *Y*-axis shows the relative absorbance in milli-absorbance units at 214 nm and the *X*-axis shows the retention time.

**Figure 4:**
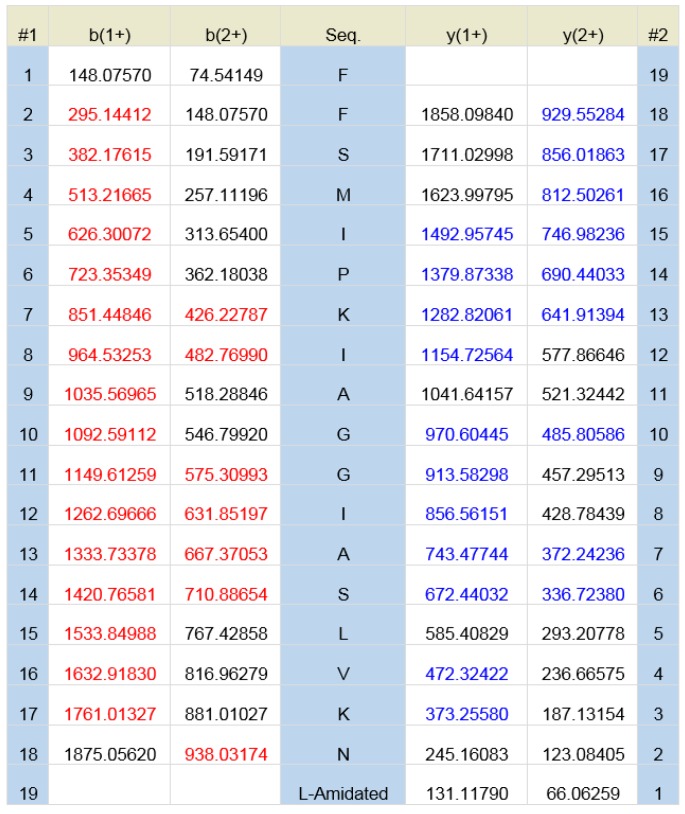
Predicted singly- and doubly-charged y-ions and b-ions arising from MS/MS fragmentation of Phylloseptin-PBa. Actual fragment ions observed following MS/MS fragmentation are indicated in blue and red.

The elution position of Phylloseptin-PBa is shown on the RP-HPLC chromatogram of the skin secretion of *Phyllomedusa baltea* (Figure 3). The peptide in the indicated fraction with a mass coincident with the approximate molecular mass of Phylloseptin-PBa from its cloned precursor, was further analyzed by MS/MS fragmentation sequencing (Figure 4). The amino acid sequence of the mature peptide was thus unequivocally identified and the glycine (G) residue in the terminal position of the Phylloseptin-PBa precursor was also confirmed as a donor for *C*-terminal amidation.

### 2.3. Peptide Synthesis

**Figure 5:**
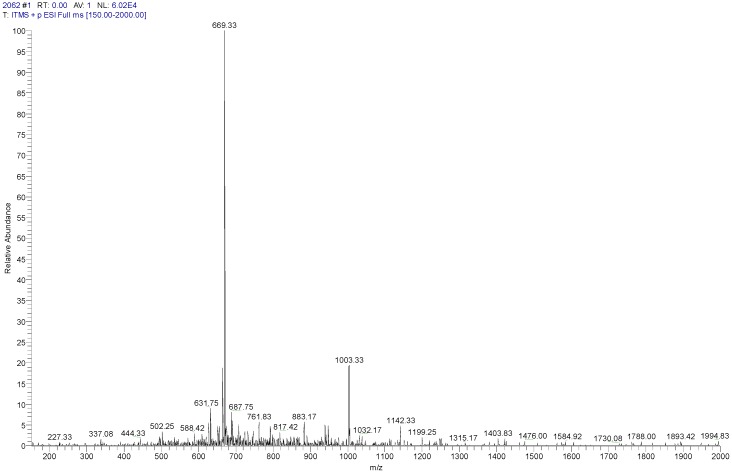
The MS full scan of synthetic phylloseptin-PBa obtained from the LCQ Fleet electrospray ion-trap mass spectrometer. The ions of m/z 669.33 and 1003.33 were triply charged and doubly charged, respectively.

The solid-phase Fmoc peptide synthesis of Phylloseptin-PBa was found to be straightforward and deemed to be highly successful. The observed molecular weight of Phylloseptin-PBa was 2004.66 Da (Figure 5). Purification of the crude product was achieved by reverse phase HPLC. Once the purified peptide was obtained, it was subjected to full ms scan and tandem mass spectrometry peptide fragmentation (MS/MS fragmentation), in order to confirm its primary structure and thus identity within the natural peptide.

### 2.4. Secondary Structure Prediction of the Peptide

**Figure 6:**
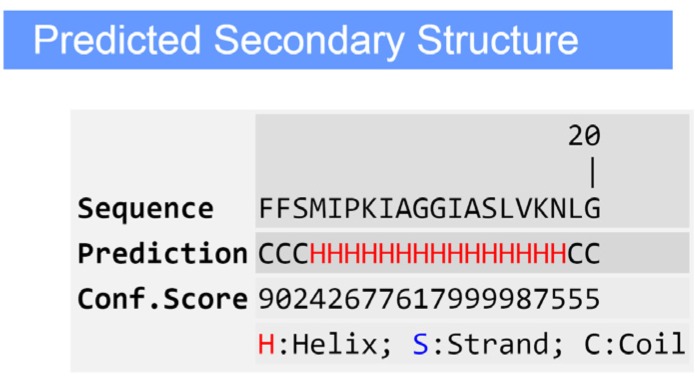
Predicted secondary structure of Phylloseptin-PBa using the on-line protein secondary structure prediction tool, I-TASSER.

**Figure 7:**
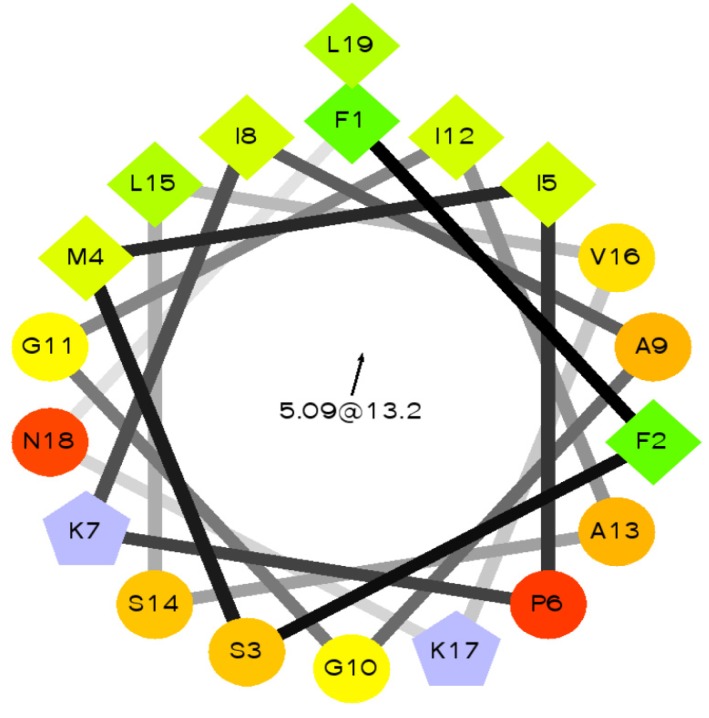
Helical wheel plot of Phylloseptin-PBa. An amphipathic character was observed with side chains of the hydrophobic residues (M4, L15, I8, F1, L19 I12, I5, V16, A9) partitioning to one side of the molecule and the cationic amino acid groups (α-amino of G10, side-chain groups of amino acids K7 and K17) appearing on the opposite side.

The predicted secondary structure of Phylloseptin-PBa, obtained through software modeling on the I-TASSER server, revealed that it contained a large proportion of α-helical domain (Figure 6). The side chains of the amino acids of the peptide partitioned into two planes, indicating their amphipathic nature. One side consisted of hydrophobic residue side chains within the helix axis, while the hydrophilic residue side chains were located on the opposite side. A helical wheel projection revealed that the peptide had a typical propensity for α-helix formation often found in typical AMPs (Figure 7).

### 2.5. Antimicrobial and Haemolytic Activity of Phylloseptin-PBa

**Figure 8:**
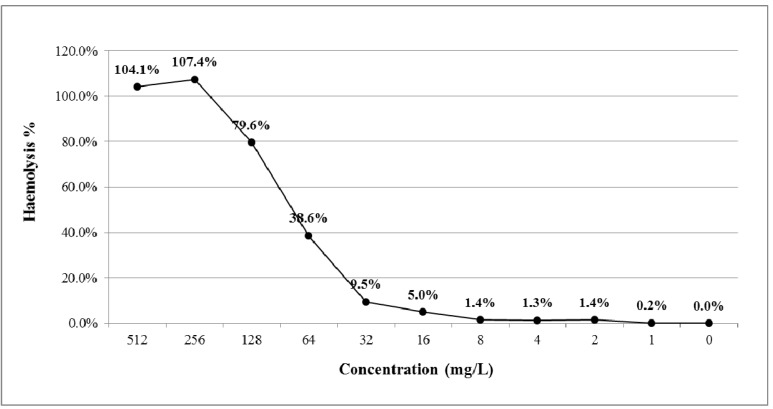
Haemolytic activity of Phylloseptin-PBa. Percentage of haemolysis was calculated in comparison to the positive control using TritonX-100.

MICs, minimal bactericidal concentrations (MBCs) and haemolytic effects of Phylloseptin-PBa were assessed. Their effects on the Gram-positive bacterium, *Staphylococcus aureus*, the Gram-negative bacterium, *Escherichia coli*, and the yeast, *Candida albicans,* are shown in Table 1. Phylloseptin-PBa exhibited potent antimicrobial activity against *Staphylococcus aureus* and *Candida albicans*. More specifically, Phylloseptin-PBa possessed the same MIC values of 8 mg/L against both organisms, these values being identical to their MBCs (Table 1). Meanwhile, Phylloseptin-PBa showed much weaker antimicrobial activity against *Escherichia coli* with an MIC value of 128 mg/L. However, Phylloseptin-PBa was associated with a relatively low cytotoxicity (1.4%) on horse erythrocytes at a concentration of 8 mg/L, but this rose to around 80% at a concentration of 128 mg/L (Figure 8).

**Table 1:** Minimal inhibitory concentrations (MIC) and minimal bactericidal concentrations (MBC) values of Phylloseptin-PBa against the Gram-positive bacterium, *Staphylococcus aureus*, the Gram-negative bacterium, *Escherichia coli*, and the yeast, *Candida albicans*.

Peptide Name	MIC (mg/L)			MBC (mg/L)		
	*S. aureus*	*E. coli*	*C. albicans*	*S. aureus*	*E. coli*	*C. albicans*
**Phylloseptin-PBa**	8	128	8	8	>512	8

### 2.6. Anti-Proliferative Effects of Phylloseptin-PBa on Human Cancer Cells

**Figure 9:**
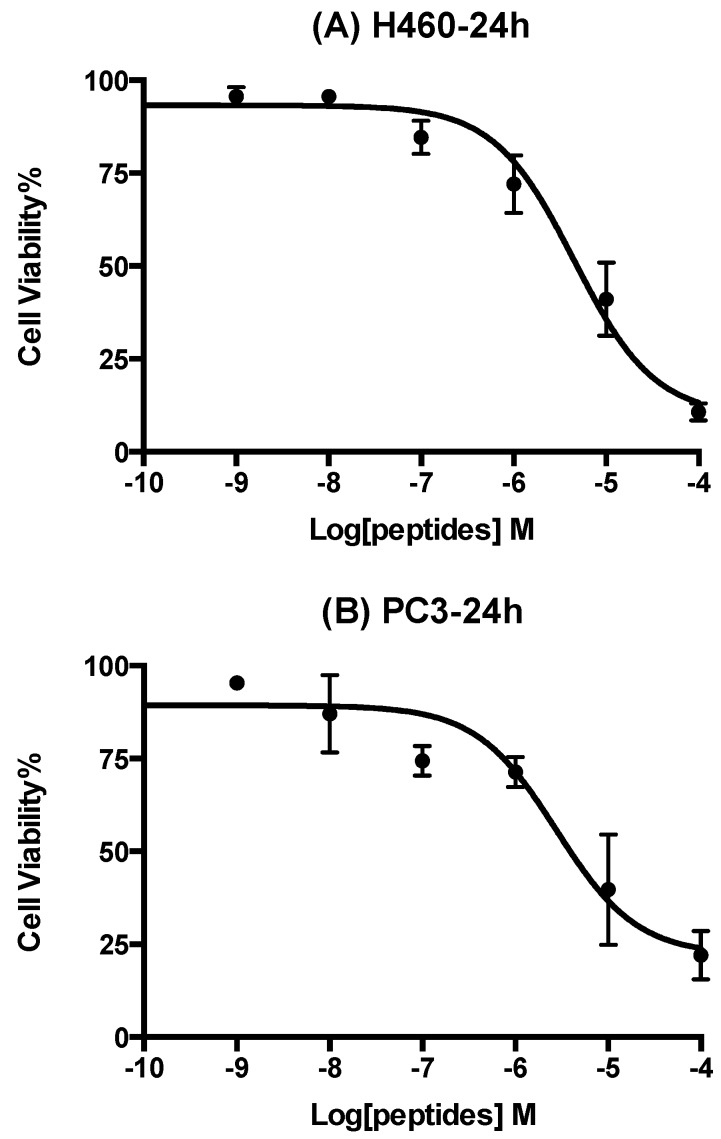

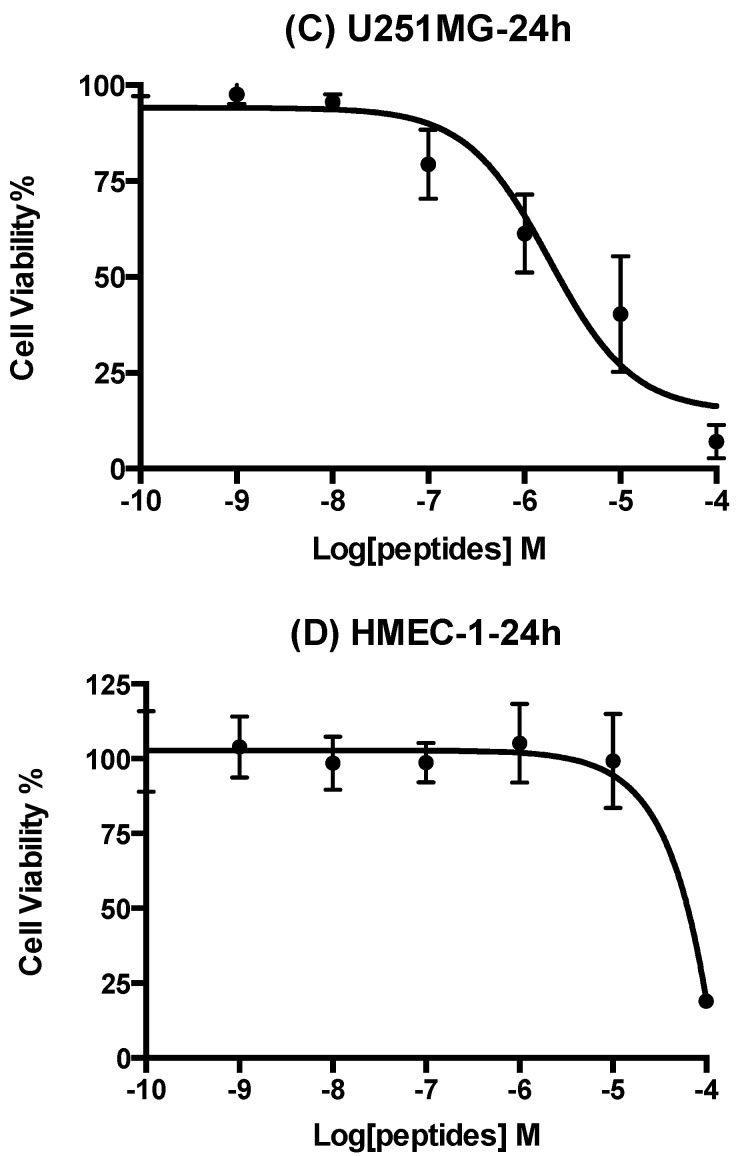
Dose-response curves of Phylloseptin-PBa on human cancer cell lines after a 24 h incubation. Panels **A**, **B**, **C** and **D** show the peptide effects on H460 (lung cancer), PC3 (prostate cancer), U251MG (neurospongioma), and HMEC-1 (microvessel endothelial cells). IC_50_ values were 4.3 µM, 2.9 µM, 1.8 µM and 36.6 µM, respectively.

Eleven human cancer cell lines were used to examine the anti-proliferative effects of Phylloseptin-PBa and they showed obvious activity in three of these: the lung cancer cell line (H460), the prostate cancer cell line (PC3) and the neurospongioma cell line (U251MG), respectively. The human microvessel endothelial cell line (HMEC-1) was used to evaluate inherent cytotoxicity of Phylloseptin-PBa against normal human cells. It showed selective cytotoxicity against the three different cancer cell lines and lower cytotoxicity against HMEC-1 cells (Figure 9). The IC_50_ values after a 24 h incubation, were 4.3 µM, 2.9 µM, 1.8 µM and 36.6 µM, respectively.

## 3. Experimental Section

### 3.1. Specimen Acquisition

Specimens of *Phyllomedusa baltea* were collected in Peru by PeruBiotech E.I.R.L. The skin secretion (40 mg lyophilised dry weight) of captured adults was subsequently harvested using mild electrical stimulation of the dorsal skin surface. Briefly, the moistened skin was stimulated by platinum electrodes (6 V DC; 4 ms pulse-width; 50 Hz) for two periods of 20 s duration. After this, stimulated secretion was collected by washing from the frog skin using distilled, deionised water. These skin secretions were snap-frozen in liquid nitrogen, lyophilised and stored at −20 °C prior to analysis.

### 3.2. Shotgun Cloning of Phylloseptin-PBa Precursor-Encoding cDNA

Five mg of lyophilised *Phyllomedusa baltea* skin secretion were dissolved in 1 mL of cell lysis/mRNA stabilisation buffer (Dynal, Merseyside, UK). Magnetic oligo-dT beads were used to isolate polyadenylated mRNA in accordance with the manufacturer’s description (Dynal, Merseyside, UK) and the isolated mRNA was subsequently subjected to RACE procedures to acquire full-length prepropeptide nucleic acid sequence data by using a SMART-RACE kit (Clontech, Oxford, UK) essentially as outlined by the manufacturer. Briefly, the 3′-RACE reactions employed a UPM primer (supplied with the kit) and degenerate sense primer (5′-ACTTTCYGAWTTRYAAGMSCARABATG-3′) that were designed to highly-conserved segments of the signal peptides of cDNAs cloned previously from other *Phyllomedusa* frogs within our group [7]. The PCR cycling procedure was as follows: step one: initial denaturation: 90 s at 94 °C; 35 cycles: denaturation 30 s at 94 °C; step two: primer annealing for 30 s at 58 °C; step three; extension for 180 s at 72 °C. PCR products were gel-purified and cloned using a pGEM-T vector system (Promega Corporation, Southampton, UK) and sequenced using an ABI 3100 automated sequencer (Applied Biosystems, Foster City, CA, USA).

### 3.3. Identification and Structural Analysis of Phylloseptin-PBa

Five mg of lyophilised skin secretion from *Phyllomedusa baltea*, were dissolved in 0.5 mL of TFA/water and cleared of microparticulates by centrifugation (2500× *g* for 5 min). The clear supernatants were pumped onto an analytical reverse phase HPLC Jupiter C5 column (250 mm × 4.6 mm, Phenomenex, UK). Peptides were eluted from the HPLC column with a linear gradient formed from 0.05/99.95 (*v*/*v*) TFA/water to 0.05/19.95/80.00 (*v*/*v*/*v*) TFA/water/acetonitrile over 240 min at a flow rate of 1 mL/min. A Cecil CE4200 Adept gradient reverse phase HPLC (Cecil, Cambridge, UK) was used and automatic collection of fractions was performed at 1 min intervals. The calculated molecular masses of predicted novel mature peptides from open-reading frames of cloned cDNAs were used to interrogate a mass spectral library of skin secretion peptides from reverse phase HPLC fractions using MALDI-TOF mass spectrometry (Perseptive Biosystems, MA, USA) in positive detection mode. The fraction containing a peptide with a molecular mass identical to that of the deduced novel cDNA-encoded peptide was analysed by MS/MS fragmentation sequencing using an LCQ-Fleet electrospray ion-trap mass spectrometer (Thermo Fisher Scientific, San Francisco, CA, USA).

### 3.4. Solid-Phase Peptide Synthesis of Phylloseptin-PBa

Following confirmation of the unequivocal primary structure of this novel peptide, it was chemically-synthesised by solid phase Fmoc chemistry using a PS4 automated solid-phase synthesiser (Protein Technologies, Inc., Tucson, AZ, USA). All of the dry amino acids were weighed and mixed with 2-(1H-benzotriazol-1-yl)-1,1,3,3-tetramethyluronium hexafluorophosphate (HBTU) activator and added to the reaction vessel on the PS4 machine. Deprotection of the Fmoc groups from the amino acids was performed in 20% piperidine in dimethylformamide (DMF). The coupling of peptide bonds was performed in 1 M N-Methylmorpholine (NMM) in DMF. After the reaction was complete, the peptide-resin mixture was washed by 30 mL of degassed dichloromethane (DCM) and subsequently dried in a vacuum desiccator overnight. Peptide was cleaved from the resin using 95% trifluoroacetic acid (TFA), 2.5% triisopropylsilane (TIPS) and 2.5% water. The confirmation of structure of the synthetic peptide was accomplished using both reverse-phase HPLC and electrospray mass spectrometry. This also established its degree of purity.

### 3.5. Antimicrobial Assays

Antimicrobial activity of the synthetic peptide was evaluated by means of determining MICs using standard strains of Gram-negative bacteria, Gram-positive bacteria and pathogenic yeast. The peptide was prepared in a concentration range of 1–512 mg/L and organisms were grown in Mueller-Hinton broth (MHB) for 18 h. Peptide solutions were then mixed with microorganism cultures (10^5^ colony forming units (CFU)/mL) and placed into 96-well microtitre cell culture plates. Plates were incubated for 18 h at 37 °C in an orbital incubator. Subsequently, the growth of bacteria/yeast was assessed by determination of optical density (OD) at λ550 nm. The MIC values were obtained according to the lowest concentrations of peptide at which no growth was detectable. Subsequently, 10 µL of each clear solution was inoculated onto Mueller Hinton agar (MHA) plates. After 24 h, the minimum bactericidal concentrations (MBCs) were obtained, based on the definition of MBC as the concentration of peptide in which colonies grew.

### 3.6. Haemolysis Assays

A 4% (*v*/*v*) suspension of red blood cells was prepared from defibrinated horse blood (TCS Biosciences Ltd, Buckingham, UK) by repeated washings and centrifugations in sodium phosphate-buffered saline (PBS). Peptide solutions of different concentrations were prepared according to the description in the antimicrobial assay in a previous section. Horse red blood cell suspension samples (200 µL) were incubated with a range of peptide concentrations, similar to the antimicrobial activity assays, at 37 °C for 60 min and 120 min. Lysis of red cells was detected by measurement of supernatants using an ELISA plate reader (Biolise BioTek EL808, Winooski, VT, USA) with optical density set at λ550 nm. Positive controls consisted of a 2% (*v*/*v*) red cell suspension and an equal volume of PBS containing 2% (*v*/*v*) of the non-ionic detergent, Triton X-100 (Sigma Aldrich, St. Louis, MO, USA). Negative controls employed consisted of a 2% (*v*/*v*) red cell suspension and PBS in equal volumes. The percentage of haemolysis was computed by the following formula:

% Haemolysis = (A – A0) / (Ax – A0) × 100%
(1)

where A is the OD (λ570) for the mixture of peptide and suspensions, Ax is OD (λ570) for the positive control and A0 is OD (λ570) for the negative control.

### 3.7. Cells Lines and Cell Culture

The human breast cancer cell lines (MB231, MB435s, MCF-7), the human prostate cancer cell lines (DU145, PC3, LNCap), the human lung cancer cell lines (H838, H460 and H157), and the human neuropongioma cell line (U251MG), were separately cultured employing RPMI-1640 culture medium (Invitrogen, Paisley, UK), or Dulbecco’s Modified Eagle’s Medium (DMEM) (Sigma, St. Louis, MO, USA), with 1% penicillin streptomycin solution (Sigma) and 10% fetal bovine serum (FBS) (Sigma) added. The human microvessel endothelial cell (HMEC-1) was employed to evaluate the cytotoxicity of the peptide against normal human cells, and these cells were grown in 10% FBS, 10 ng/mL EGF, 10 mM L-Glutamine, 1% penicillin streptomycin supplemented MCDB131 medium (Gibco, Paisley, UK). The selected cells were inoculated into 90 mm culture dishes (Nunc, Roskilde, Denmark) or into 75 cm^2^ culture flasks (Nunc). Following this, flasks were placed in an incubator with a humidified environment containing 5% CO_2_.

### 3.8. Assessment of Cancer Cell Antiproliferative Activity Using the MTT Cell Viability Assay

Cancer cell line proliferation and viability were assessed using the MTT cell viability assay [8]. Briefly, each of the cancer cell lines was seeded at a density of 5 × 10^3^ cells per well onto 96 well plates. Following this, cell lines were prepared with gradient concentrations of peptide and incubated over 24 h. After this, 10 µL of 5 mg/mL yellow coloured MTT solution (Sigma) were added to all wells and incubated again for 4 h. Once the supernatants were removed by a syringe, 100 µL of DMSO were added to all wells after gently agitating in order to completely mix the formazan crystals that had developed. A Synergy HT plate reader (BioTek, Winooski, VT, USA) was set at 550 nm for recording the absorbance, and the statistical analysis of results was performed using Student’s *t*-test through GraphPad Prism 5.0 software. The final results were considered to be statistically significant if the *p* value was ˂0.05.

## 4. Discussion

Phylloseptins, a new family of antimicrobial peptides, has been recently described as having primary structures containing 19–21 amino acid residues. Family members share similar structural attributes including their *N*-terminal regions and *C*-terminal amidation [6]. Phylloseptin-PBa reported here, was found to be structurally related to earlier reported phylloseptins. The first domain of the precursor protein encoded a 22 amino acid residue signal peptide and this displays around 90% similarity across the family [6,9]. These signal peptides were characterized by the conservation of most residues in the *N*-terminal sequence: Met-Ala-Phe-Leu-Lys-Lys-Ser-Leu-Phe-Leu-Val-Leu-Phe-Phe/Leu-Gly-Leu-Val-Ser-Leu-Ser-IIe-Cys-. The following 24 amino acids encode an acidic amino acid residue-rich spacer domain which, although its function has not been fully explained, may have a role to play in peptide storage or packaging within secretory cells [5]. The third domain contained another highly conserved classical -Lys-Arg-(-K-R-) propeptide convertase cleavage site. The fourth domain encoded the mature peptide and consisted of 19 amino acids. It would appear that the length of these peptides plays an important role in their actions. Like other phylloseptins, Phylloseptin-PBa also displayed a proline at position 6 that affected the linearity of the structure [9]. The *C*-terminal residue of Phylloseptin-PBa was the glycyl residue amide donor for this post-translational modification, which is quite a common modification in many antimicrobial peptides. This amidation is thought to enhance the net positive charge increasing the cationicity of the peptide, which would lead to enhancement of the antimicrobial effect [8]. Based on the clearly identified structure of Phylloseptin-PBa, whose structure was initially deduced from cloned skin cDNA of the frog, *Phyllomedusa baltea*, there was clear structural similarity to homologues from *Phyllomedusa* *sauvagei*, *Phyllomedusa* *hypochondrialis*, *Phyllomedusa* *azurea* and *Phyllomedusa* *bicolor* [4]. Many frogs produce antimicrobial peptides that have overlapping structural features. Despite the fact that Phylloseptin-PBa was very similar in primary structure to other members of the phylloseptin family with the same number of amino acid residues and positive charge, secondary structure prediction analysis found that Phylloseptin-PBa had a higher helical content and hydrophobicity [8]. Several researchers have reported that hydrophobicity can enhance the interaction between peptides and bacterial membranes. On the other hand, it could also result in increasing mammalian cell cytotoxicity [10].

The result of the antimicrobial assays revealed that Phylloseptin-PBa produced growth inhibition on all three typical microorganisms tested. It had more potent antimicrobial activity on the yeast and Gram-positive bacterium, but only had weak effects on the Gram-negative bacterium. More specifically, the results showed that Phylloseptin-PBa possessed the same MIC value for *C. albicans* and *S. aureus*. However, the MIC value of Phylloseptin-PBa on *E. coli* showed a low potency against this organism. The higher potency of Phylloseptin-PBa against *S. aureus,* when compared with *E. coli,* could be explained by the fact that cells of the former possess a single bilayer lipid membrane (BLM), while Gram-negative bacteria have a rich acidic membrane that reduces the charge on their surfaces [11,12]. This was an important observation in that Phylloseptin-PBa might be of use in disseminated Gram-positive bacterial infections. Haemolytic activity of AMPs is an undesirable side-effect as reported by Zhang et al [13]. According to structure/activity relationships, one of the important features of AMPs is the proportion of their hydrophobic amino acid residues, which normally constitute at least 50% of their entire amino acid sequences [14,15,16]. Hydrophobicity not only increases the potency of antimicrobial peptides, killing bacteria via membrane disruption, but also enhances the possibility of interaction with mammalian zwitterion membranes, which are less negatively charged than bacterial membranes and have a higher content of cholesterol [14,15,16]. Similar to many other antimicrobial peptides, Phylloseptin-PBa contained over 50% of hydrophobic amino acid residues, which resulted in a relatively high haemolytic effect at high concentrations but was very low at MICs for *S. aureus* and *C. albicans*.

A growing number of researchers have shown that some antimicrobial peptides possess both antimicrobial and anticancer activity, although the anticancer mechanism of these peptides is not clearly known as yet. However, previous studies have indicated that antimicrobial peptides kill cancer cells by cellular membrane lysis [17]. There are many structural similarities among these peptides, mostly with regard to their cationicity and amphipathic nature. These common features have illustrated that membrane interaction is crucial for the disrupting effect of these peptides, via rapidly binding with the plasma membrane of cancer cells producing cell death. Therefore, the conclusions of these researchers have supported the “carpet” and “barrel-stave” mechanisms for membrane lysis [14]. This may provide another option to traditional anticancer chemotherapies, without strong side effects and low possible development of multi-drug resistance, making antimicrobial peptides excellent candidates for novel anticancer therapy [18].

Anti-proliferative activity of Phylloseptin-PBa was examined by use of MTT cell viability assays using eleven human cancer cell lines. Data showed that Phylloseptin-PBa had a broad-spectrum of anticancer activity with but with selectivity in inhibiting different categories of cancer cell lines. Compared to other cell lines, this peptide was more potent in inhibiting the growth of H460, PC3 and U251MG cells. In addition, this peptide also possessed anti-proliferative activity for HMEC-1 cells at the highest concentration of 0.1 mM in the MTT assay. This sensitivity bias of different cell lines with the same peptide may be due to the different structures and components of cell membranes. Many studies have found that cancer cells can over express O-glycosylated mucins and high molecular mass glycoproteins on the cell membrane, which can provide additional negative charges [17,18,19]. However, in common with the antimicrobial mechanisms of AMPs, their anticancer mechanisms are still unclear. Why the innate immune system produces peptides with multiple functions is also still unknown. Nevertheless, with further studies in this area, more details of AMP action will be discovered in order to elucidate their mechanisms. More significantly, cationic antimicrobial peptides have outstanding advantages over conventional drugs which include their broad-spectrum activity, rapid killing of cancer cells and obviation of conventional chemotherapy resistance. These factors herald the possibility that AMPs may become a new option of treatment for cancer patients.

Recently, it has been discovered that phylloseptin peptides not only possess broad-spectrum antibacterial and antifungal activities but also have the ability to release insulin to improve glucose tolerance in mice. More specifically, this has been achieved using a synthetic replicate of the peptide, phylloseptin-L2, on stimulating basal insulin secretion rates in BRIN-BD11 cells. Thus, as there is a potential for developing these peptides into agents for treating Type 2 diabetes, there is understandable interest in isolation and characterization of members of this peptide family [6,20].
